# Trajectory Inference with Cell–Cell Interactions (TICCI): intercellular communication improves the accuracy of trajectory inference methods

**DOI:** 10.1093/bioinformatics/btaf027

**Published:** 2025-02-03

**Authors:** Yifeng Fu, Hong Qu, Dacheng Qu, Min Zhao

**Affiliations:** School of Computer Science & Technology, Beijing Institute of Technology, Beijing 100081, China; Center for Bioinformatics, State Key Laboratory of Protein and Plant Gene Research, School of Life Sciences, Peking University, Beijing 100871, P.R. China; School of Computer Science & Technology, Beijing Institute of Technology, Beijing 100081, China; Information Center, China Association for Science and Technology, Beijing 100863, P.R. China; School of Science, Technology and Engineering, University of the Sunshine Coast, Maroochydore DC, QLD 4558, Australia

## Abstract

**Motivation:**

Understanding cell differentiation and development dynamics is key for single-cell transcriptome analysis. Current cell differentiation trajectory inference algorithms face challenges such as high dimensionality, noise, and a need for users to possess certain biological information about the datasets to effectively utilize the algorithms. Here, we introduce Trajectory Inference with Cell–Cell Interaction (TICCI), a novel way to address these challenges by integrating intercellular communication information. In recognizing crucial intercellular communication during development, TICCI proposes Cell–Cell Interactions (CCI) at single-cell resolution. We posit that cells exhibiting higher gene expression similarity patterns are more likely to exchange information via biomolecular mediators.

**Results:**

TICCI is initiated by constructing a cell-neighborhood matrix using edge weights composed of intercellular similarity and CCI information. Louvain partitioning identifies trajectory branches, attenuating noise, while single-cell entropy (scEntropy) is used to assess differentiation status. The Chu–Liu algorithm constructs a directed least-square model to identify trajectory branches, and an improved diffusion fitted time algorithm computes cell-fitted time in nonconnected topologies. TICCI validation on single-cell RNA sequencing (scRNA-seq) datasets confirms the accuracy of cell trajectories, aligning with genealogical branching and gene markers. Verification using extrinsic information labels demonstrates CCI information utility in enhancing accurate trajectory inference. A comparative analysis establishes TICCI proficiency in accurate temporal ordering.

**Availability and implementation:**

Source code and binaries freely available for download at https://github.com/mine41/TICCI, implemented in R (version 4.32) and Python (version 3.7.16) and supported on MS Windows. Authors ensure that the software is available for a full two years following publication.

## 1 Introduction

Investigating cellular differentiation and developmental dynamics is a key research area in cancer cell biology (Fares *et al.* 2020). Single-cell isolation and RNA sequencing technologies have pivotal roles in this field, serving as vital biological tools uncovering fundamental metastatic processes, elucidating cancer immune microenvironments, and understanding gene roles (Binnewies *et al.* 2018). The advent and continual advancement of commercialized single-cell RNA sequencing (scRNA-seq) platforms have provided large-scale insights on cell differentiation and development processes at single-cell levels (Baysoy *et al.* 2023). A crucial task in this pursuit is inferring cell differentiation trajectories based on scRNA-seq data, with a view to comprehending evolving cell patterns in diverse cancers.

Currently, a multitude of bioinformatics algorithms (>100) are being used to infer cell trajectories. Among the most recognized are Monocle (Trapnell *et al.* 2014), TSCAN (Ji and Ji 2016), and partition-based graph abstraction (PAGA) (Wolf*et al.* 2019). These methods leverage gene expression similarities between cells to construct cell lineage models, arranging cells along differentiation paths to reconstruct cell differentiation and development processes. Each cell is assigned an attribute called “pseudotime,” which quantifies a cell’s position in developmental processes. Cells with lower pseudotime values are positioned earlier in processes. However, such algorithms face challenges, including high scRNA-seq data dimensionality, substantial noise, and a requirement for users to have certain biological information on the datasets to effectively use the algorithms. In contrast, while intercellular communication information is a crucial regulatory factor in cell differentiation and developmental processes (Camp *et al.* 2017, Kyaw *et al.* 2023), contemporary algorithms inferring cell trajectories often overlook and under-investigate such information.

Intercellular communications, facilitated by biomolecular mediators, are indispensable for cell development, orchestrating cell differentiation and sculpting the intricate structures required by organisms. Cell–cell interactions (CCI) in cancer occur via various mechanisms, including direct contact, soluble factor secretion (e.g. cytokines, chemokines, and growth factors), and extracellular vesicles. These mechanisms regulate processes such as angiogenesis, immune evasion, metabolic reprogramming, and the generation of a supportive tumor microenvironment (Liu and Zhao 2021). Recent studies have identified specific cell communication patterns induced by cytokines (Liu *et al.* 2023) and neuropeptide-receptor networks (Liu and Zhao 2021) across multiple cancer types, highlighting intercellular signaling in tumor development and progression.

Despite the significance of CCI in cancer development, there is a dearth of cell trajectory inference tools using intercellular communication information for differentiation analysis. Some studies have uncovered intercellular communication patterns. Armingol *et al.* (2022) confirmed that cell-to-cell interactions were negatively correlated with the spatial distance between cells. In addition, Zeira *et al.* (2022) confirmed that spatially adjacent cells had more similar gene expression characteristics. From these studies, we posit that during differentiation and development, cells exhibiting higher gene expression similarity patterns are more prone to information exchange via biomolecular media, such as cytokines and neurotransmitters. Consequently, we have introduced CCI information, delineating intercellular communications at single-cell resolution. CCI addresses potential issues in scRNA-seq data, such as sampling and measurement issues. We have developed a novel Trajectory Inference with Cell–Cell Interaction (TICCI) method, leveraging intercellular communication information examining cell trajectories in scRNA-seq data. By incorporating CCI information, we enhance biological insights from inferred trajectories.

## 2 Materials and methods

### 2.1 Method overview

**Figure 1.**
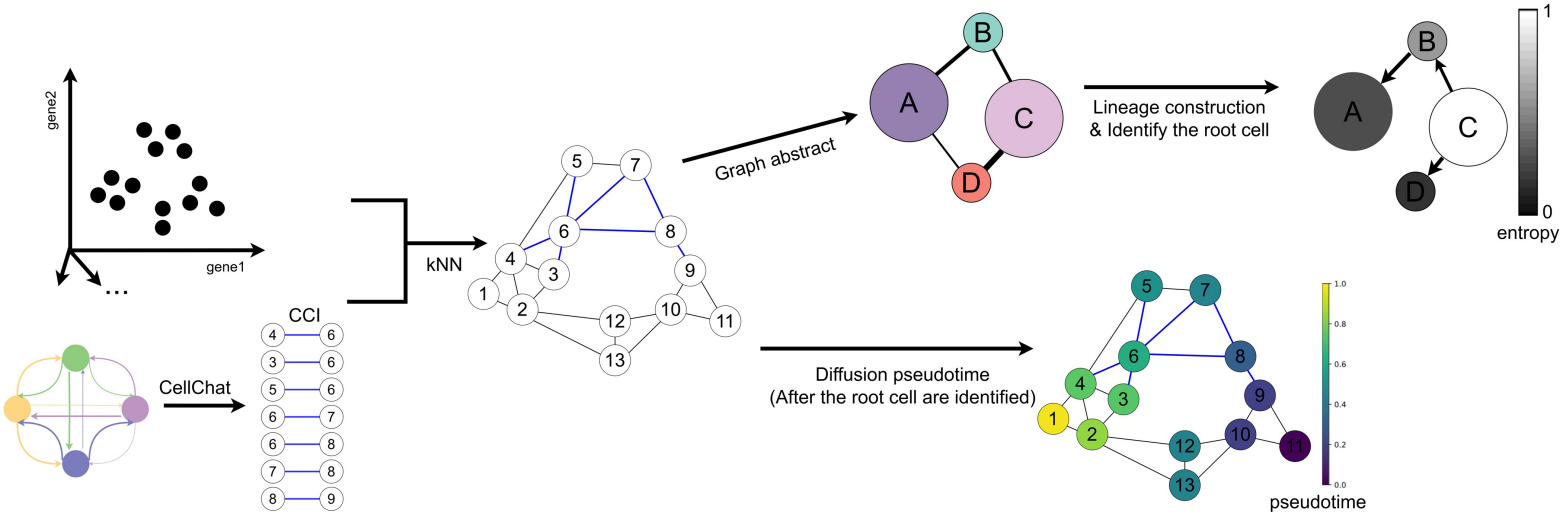
Schematic showing the TICCI algorithm workflow. The algorithm starts with single-cell RNA sequencing (scRNA-seq) data. First, cells are clustered and communication patterns between cell clusters calculated by CellChat (cell communication analysis tool), followed by communication result processing into CCI information that represents intercellular communication information at a single-cell resolution. Next, CCI information is synthesized to construct a class KNN graph on downscaled gene expression data, and partition clustering and image abstraction are performed to generate cell topology. After calculating stable state single-cell Entropy (scEntropy) in cell partitions, a maximum directed spanning tree is generated on the class KNN graph (as a genealogical model). The stable state scEntropy of each partition is determined by scEntropy in cells in the partition, which is used to measure cell differentiation status. The genealogical branch is then directed from the partition with higher stable state scEntropy to a partition with a lower stable state scEntropy. Finally, the starting cell is identified as the cell with the highest scEntropy in the partition with the highest stable state scEntropy, and diffusion pseudotime is used to calculate the pseudotime for each cell.

To develop a cell trajectory inference method incorporating intercellular communication data, we developed the TICCI algorithm (Fig. 1). The algorithm has four main steps: (i) Computing CCI information for intercellular communications between cells based on molecular ligand receptors. (ii) Cell clustering partitioning and graphical abstraction. (iii) Calculating stable state single-cell Entropy (scEntropy) in each cell partition and inferring a genealogical cellular model. (iv) Calculating the pseudotime for each single cell.

A workflow (Fig. 1) depicts the five key stages of the TICCI algorithm:

1. TICCI constructs a cell neighborhood matrix with edge weights based on intercell union probabilities and CCI information to quantify intercell similarity.
2. TICCI clusters and partitions cells using the Louvain partitioning algorithm to identify trajectory branches in terms of coarse-grained Louvain partitions, thereby reducing noise in scRNA-seq data.
3. TICCI uses scEntropy to assess differentiation status in partitions and automatically determines the genealogical model independent of external bioinformation.
4. TICCI uses the Chu–Liu algorithm to construct a directed least-square partitioning map on the partition graph, identifying trajectory branches via directed minimum spanning trees.
5. TICCI computes cell pseudotimes using a modified diffusion pseudotime algorithm, applicable to nonconnected trajectory topologies.

### 2.2 Computing CCI information

Most cell trajectory inference methods focus on scRNA-seq data analyses, but they are limited in detecting potential intercellular communications, which often leads to heterogeneity and cell state transitions, as previously demonstrated (Camp *et al.* 2017). TICCI proposes a CCI matrix to represent intercellular communication information at single-cell resolution and add it to trajectory inference.

First, TICCI computes communication patterns between cell subpopulations, and for datasets without cell classification, TICCI uses the k-means method (Arthur and Vassilvitskii 2007) to unsupervisedly classify cells into groups, with cluster numbers determined by the Gap statistic. We use the R package “cluster” (https://cran.r-project.org/web/packages/cluster) to implement k-means clustering and compute the Gap statistic. We use the R package “CellChat” (https://github.com/sqjin/CellChat) to calculate intercellular communication patterns among cell subpopulations. CellChat enables the quantitative inference and analysis of intercellular communication networks from a scRNA-seq data tool, as proposed by Jin *et al.* (2021). Compared to previous methods, CellChat probes communication patterns in multiple ligand/receptor gene pairs and predicts abundant signaling interactions between two cell populations. Specifically, Jin *et al.* first developed CellChatDB, a database containing mouse and human ligand–receptor interactions. For a given scRNA-seq dataset, CellChat first identifies signaling genes that are differentially expressed between cell groups, and then models ligand–receptor-mediated signaling interactions using the law of mass action.

Cell communication strength is proportional to the expression of corresponding ligand–receptor genes, and genes with expression levels greater than a particular threshold are deemed validly expressed. In this study, the threshold is 1. CCI information is constructed based on communication network results between cell subpopulations. Specifically, for each set of a particular ligand–receptor pair k in communication from cell group *i* to cell group *j*, cell *p* is considered to be in communication with cell *q* if cell *p* and cell *q* satisfy the condition that *p* belongs to cell group *i* and the ligand gene *L* is validly expressed in *p*, and *q* belongs to cell group *j* and the receptor gene is validly expressed in *q*. The probability of communication between cell *i* and cell *j* at ligand–receptor pair *k* is equal to$\;C_{i,j}^{k}$. The probability of communication between cell *p* and cell *q* in a CCI,$\;P_{p,q}$, which equals the sum of the communication probabilities of all ligand–receptor pairs between the two cells, is shown:

$$P_{p,q}=\sum_{k}^{K_{i,j}}C_{i,j}^{k}$$

where$\;K_{i,j}\;$denotes all communicating ligand–receptor pairs between cell group *i* and cell *j*, and$\;p\in i,q\in j,L_{p}>1,R_{q}>1$, The set K represents the collection of all ligand–receptor pairs k.

### 2.3 Cell clustering partitioning and graph abstraction

Previous trajectory inference methods usually rely only on gene expression matrices to quantify similarities between cells. However, these methods may fail to adequately capture inter-cell interaction complexity, which we address with CCI information.

First, to identify gene expression similarities between cells in a given scRNA-seq dataset, TICCI uses Principal Component Analysis (PCA) to dimensionally reduce the gene expression matrix. PCA is a linear dimensionality reduction method used to project data onto the first x coordinates with higher variance, which is then used to reduce dataset dimensionality while maintaining dataset features that contribute the most to variance (Maćkiewicz and Ratajczak 1993). TICCI then uses Nearest-Neighbor-Descent in Uniform Manifold Approximation and Projection (UMAP, a dimensionality reduction technique that preserves the global structure of high-dimensional data while effectively maintaining local relationships) (Armstrong *et al.* 2021) to construct a symmetric K-Nearest Neighbors (KNN, a simple, nonparametric algorithm used for classification and regression tasks, which assigns a data point to the majority class among its k closest neighbors in the feature space)-like graph over reduced dimensionality data, where nodes represent cells in the dataset, and edge weights represent joint probabilities between two cells, which are stored in the adjacency matrix. The joint probability quantifies the degree of similarity between cells in terms of gene expression.

Similar to the KNN Search, Nearest-Neighbor-Descent calculates *k* nearest neighbor points for each cell. For a pair of neighbor cells *i* and cell *j*, the conditional probability$\;p_{i|j}\;$is calculated, i.e. the probability of cell *i* being a neighbour of cell *j* is known is shown (Armstrong *et al.* 2021):

$$p_{i|j}=e^{-\frac{d\left(xi,xj\right)-\rho_{i}}{\sigma_{i}}}$$

where$\;x_{i}\;$denotes cell *i*,$\;d\left(x_{i},y_{i}\right)\;$is the Euclidean distance between cell *i* and *j*, and$\;\rho_{i}\;$and$\;\sigma_{i}\;$are the regulation parameters.$\;\rho_{i}\;$denotes the distance between cell *i* and the nearest point, and the parameter$\;\rho_{i}\;$is added to avoid a disjointed graph situation. If cell i is far away from all cells, the probability that cell *i* will be a neighbor of all cells is close to zero. Adding$\;\rho_{i}\;$ensures that the conditional probability that at least one cell is a neighbor to cell *i* is 1, thereby guaranteeing connectivity in the graph. The parameter$\;\sigma_{i}\;$is determined by the *k* value, similar to KNN, where *k* is the number of immediate neighbors considered for each cell. The$\;\sigma_{i}\;$satisfying the condition$\;k=2^{\sum_{j}p_{ij}}\;$is obtained by a binary search, and$\;p_{ij}\;$is the joint probability of cell *i* with cell *j*. Next, to ensure connectivity symmetry between two cells, the joint probability of cell *i* and cell *j* is proposed based on conditional probability (McInnes and Healy 2018):

$$p_{ij}=p_{i|j}+p_{j|i}-p_{i|j}p_{j|i}$$

**Figure 2.**
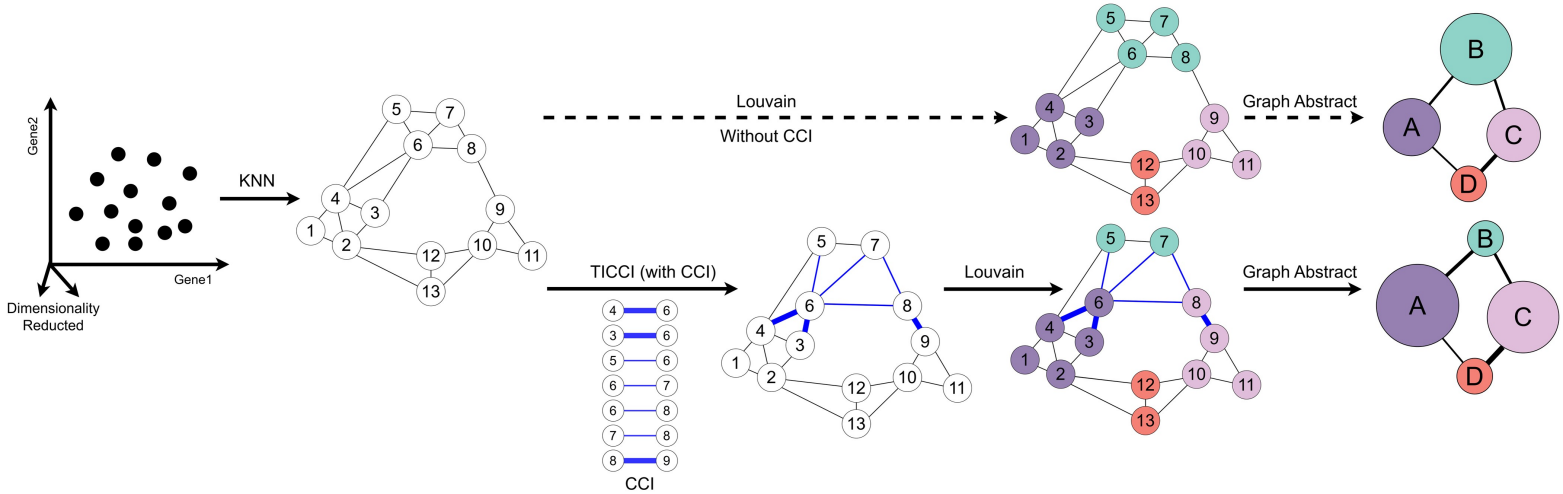
Schematic showing the CCI integration of joint probabilities representing inter-cell similarity. TICCI first constructs a symmetric class KNN graph on dimensionality-decreased data using an approximate nearest-neighbor search in the UMAP algorithm, and then adjusts edge weights to optimally represent cell similarity and changes the results of clustering partitioning and diffusion pseudotime using CCI information. The top branch of the figure represents the direct partitioned clustering process without CCI information, and the bottom represents added CCI information to optimize partitioning results as represented by different colored spheres.

Based on critical intercellular communication roles in cell development and differentiation, TICCI integrates CCI information into constructing joint probability neighborhood matrices between storage cells (Fig. 2). This enhances its accuracy in representing similar gene expression patterns so that it attenuates noise due to scRNA-seq issues and reveals richer biological information. In cell differentiation and development studies, we typically hypothesize that in interacting cell populations, cells that show greater similarity in terms of gene expression patterns are more likely to exchange information via intercellular communications. In contrast, cells that lack similar gene expression patterns are less likely to engage in such communications. Based on such assumptions, we combined intercellular association probabilities and communication probabilities to calculate new edge weights in the class KNN graph to quantify similarity degrees between cells. The formula representing similarity between cell *p* and cell *q* is defined:

$${\hat{p}}_{ij}=p_{ij}+KP_{i,j}$$

where *K* = the CCI weight parameter.

To identify cell trajectory topology, TICCI uses the Louvain algorithm to cluster cells and construct a PAGA graph (Wolf *et al.* 2019). The Louvain algorithm is a community discovery algorithm that identifies communities based on their modularity, without having prior knowledge on community numbers or size (De Meo *et al.* 2011). The basic algorithm concept is for each node to explore different community labels in its neighborhood and assign it to the community that maximizes modularity. Then, each community is treated as a new node, and the process is repeated until the modularity cannot be increased any further in order to generate optimal community partitioning. A PAGA graph is an undirected graph where nodes represent partitions and edge weights represent “PAGA connectivity measures” between two partitions; it is a test statistic which quantifies the connectivity degree of two partitions and has a close relationship with modularity. For each pair of clusters, PAGA connectivity is the ratio of the number of inter-edges between clusters, normalized to the number of inter-edges expected under random edge assignment (Wolf *et al.* 2019). We used the Python package scanpy (https://scanpy.readthedocs.io/en/latest/index.html) to implement PCA, UMAP proximity searches, and Louvain algorithms. This package allows the user to set resolution parameters for graphical abstraction. In this study, the dimensions chosen for PCA numbered 50 and the neighbors chosen for UMAP proximity searches numbered 10.

### 2.4 Lineage model construction

Different cell partitions can reveal biologically relevant heterogeneity, suggesting that cells may have different differentiation capacities. In order to not rely on additional biological information when accurately inferring cell trajectories, it is important to unsupervisedly assess the differentiation capacity of each cell partition. To this end, TICCI uses scEntropy to measure functional cell plasticity. The measurement of scEntropy is derived from cellular functional activation levels, as reflected by gene expression in individual cells, which indicates each cell’s differentiation potential. Entropy has been widely used in statistical mechanics, thermodynamics, and information theory as a measure of disorder or uncertainty in a system (Guo *et al.* 2017). Entropy is also a useful measure of cellular heterogeneity: cells with low entropy exhibit narrow, well-defined mRNA and protein expression patterns (under strict regulatory constraints), whereas high-entropy cells have broad, diverse expression patterns (under weaker regulatory constraints and therefore have multiple potentials) (MacArthur and Lemischka 2013). Specifically, Guo *et al.* first classified all genes into different functional groups, then calculated the activation probability of each functional group in a single cell, and finally calculated cell scEntropy based on the activation probability of functional groups in the cell. We used the R package “SLICE” to perform scEntropy calculations (Guo *et al.* 2017).

Since cellular differentiation is likely to transition to full maturity via a series of intermediate states, individual cells isolated at any given developmental stage may generate cell mixtures (in an asynchronous manner) at different stages, with some stable cells, while others occur in a transition state from one stable state to another. In a given scRNA-seq dataset, multiple stable states may coexist. Cells in the transitional phase have insufficient functional activation, resulting in low entropy differentiation. This reduces the distinction in entropy levels between different partitions, thereby diminishing the effectiveness of entropy-based differentiation for pseudotime ordering. To eliminate transition state cell influences on assessing the differentiation potential of each partition, TICCI was first used to calculate “stable state scEntropy” in partitions on the PAGA graph, and then determine partition positions on the differentiation path according to their “stable state scEntropy.” TICCI selects a portion of cells with the minimum entropy as the stable state core cell set for each partition. In our studies, cells with the top 25% lowest entropy in each partition were selected as a core cell set.

After determining the partition order on the differentiation path, TICCI transforms the undirected PAGA graph into a directed graph with unchanged node and edge weights. Edge direction is determined based on stable state scEntropy in partitions, with the partition with the higher entropy value pointing to the lower one. TICCI then automatically recognizes the partition with the highest entropy value as the initial differentiation lineage state and then uses the Chu–Liu algorithm (Chu 1965) to construct a directed maximal spanning tree on the directed PAGA graph to infer the differentiation lineage. This algorithm is a graph-theoretic algorithm for directed graphs that constructs a maximal spanning tree by recursively searching for augmenting paths.

### 2.5 Estimating cell pseudotimes

TICCI use modified diffusion pseudotime (DPT) to estimate cell pseudotimes. When compared to the original version, the improved DPT can be applied to cell trajectories in nonconnected topologies. DPT computes a type of random walk-based distance between two cells, which is based on the Euclidean distance in the “Diffusion Map space.” DPT improvements were proposed by Wolf and others (Wolf *et al.* 2019) to facilitate its application to nonconnected graph structures. Specifically, an infinite distance is assigned to cells in disconnected clusters, and distances between cells in connected regions of the graph are computed as in DPT.

TICCI first automatically selects a cell with the highest stable state scEntropy in the partition, with the highest stable state scEntropy as the root cell and sets its pseudotime to 0. It then iterates over all other cells, taking the random walk distance between each cell and the root cell as the pseudotime for that cell. We used the Python package scanpy to implement an improved DPT.

### 2.6 Datasets and preprocessing

We applied TICCI on two independent real datasets and two simulated datasets. Real dataset 1 consisted of 266 differentiated human skeletal muscle myoblasts (HSMMs). Real Dataset 2 consisted of 101 mouse lung alveolar type 2 (AT2) cells. ScRNA-seq data and information on cell type and developmental stage for both datasets were obtained from Guo *et al.* (2017). The HSMM dataset measured developmental processes during skeletal muscle cell differentiation, including three cell types labeled as proliferating cells, differentiating into myoblasts, and mesenchymal cells. The HSMM dataset measures developmental processes in human skeletal muscle cell differentiation, including three types of cells: proliferating cells, differentiating myoblasts, and interstitial mesenchymal cells. The AT2 dataset measures developmental processes during mouse alveolar epithelial cell differentiation, including cells at E14.5, E16.5, E18.5, and adult stages. Original quality control, cell type assignments, and other analyses performed on scRNA-seq data from these datasets are described in the relevant manuscripts (Guo *et al.* 2017). Two simulation datasets were generated by scMultiSim (Li *et al.* 2023). scMultiSim is an in-silico simulator that generates multi-modal single-cell data including cell–cell interations and other various biological factors. The parameters and preprocessing for generating the simulated dataset are documented in the Supplementary File.

## 3 Results

To verify TICCI accuracy, we successfully constructed cell trajectories on two real and two simulated scRNA-seq datasets and verified that genealogical branching and gene marker expression along cell trajectories were consistent with differentiation and developmental process in cells. This confirmed trajectory reliability by TICCI. Using real cell differentiation stage labels as a benchmark, we verified that CCI information enhanced trajectory inference method accuracy. Comparisons with other trajectory inference algorithms showed that TICCI performed well in terms of pseudotime ordering accuracy. The results for the two real datasets are shown below and the results for the two simulated datasets are presented in the Supplementary File.

### 3.1 TICCI infers HSMM trajectories

We first validated TICCI using the HSMM dataset. As in Guo *et al.* (2017), we evaluated the number of expressed genes per cell (FPKM > 1) and excluded five cell types (“T0_CT_A11,” “T0_CT_E10,” “T0_CT_C09,” “T48_CT_C02,” and “T48_CT_H04”) as their expression levels were below the three-quarters value of the 0.1 quartile. Fragments Per Kilobase of transcript per Million mapped reads (FPKM) is a standardized method for quantifying gene expression and represents the number of fragments per kilobase of transcript (i.e. mRNA) per million fragments mapped to a reference genome. We screened for genes expressed in over 5% of cells, normalized them, and performed log2 transformation analyses to enhance comparability and distinguish low and high-abundance genes. To prevent infinite values, a pseudo-count of 1 was added during log2 transformations.

**Figure 3.**
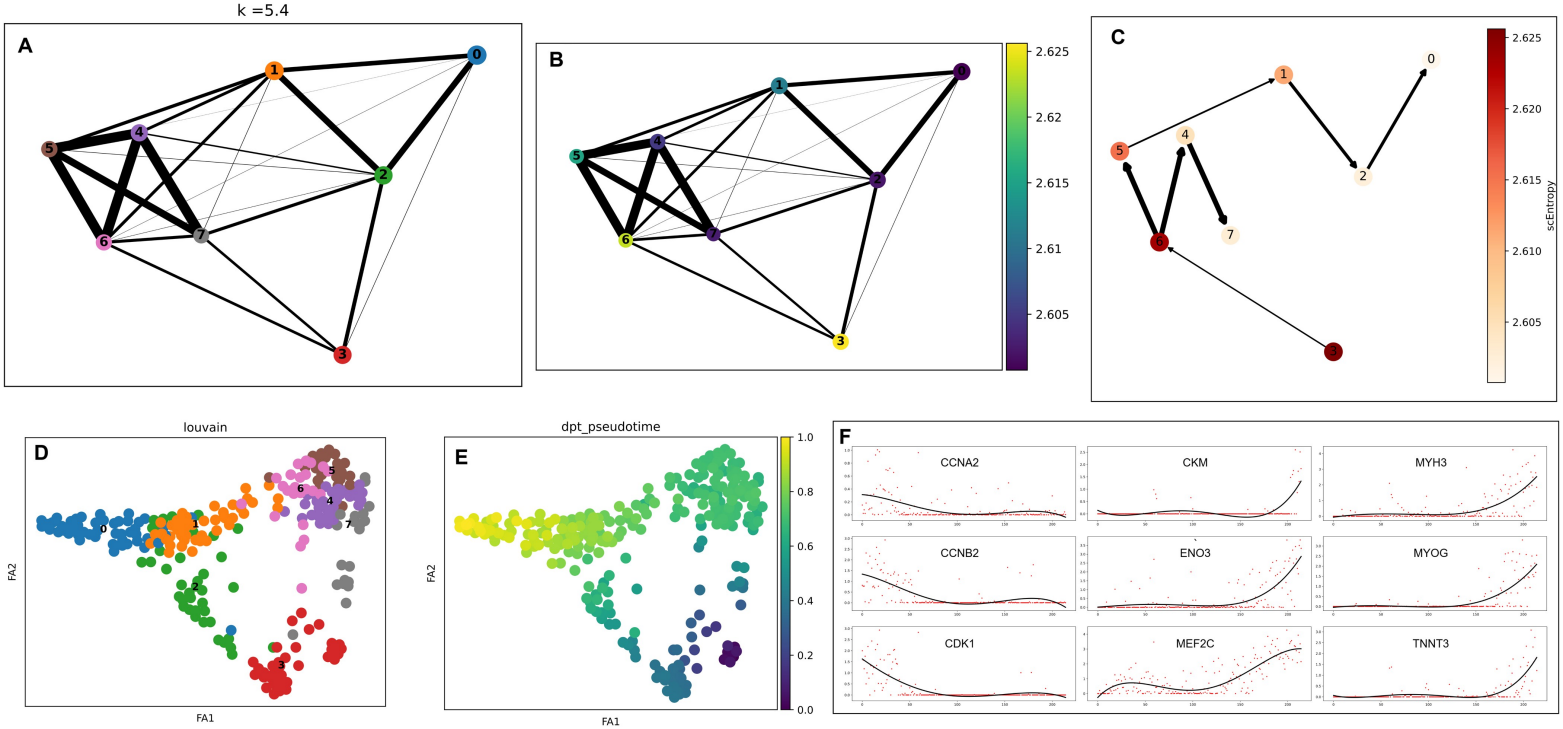
TICCI inferred trajectories in differentiated human skeletal muscle myoblasts (HSMMs). (A) TICCI at the optimal CCI weight of 5.4 abstracted HSMMs into eight partitions, with thicker connecting lines between partitions indicating higher expression similarity. (B) To determine a lineage model, TICCI calculated stable state scEntropy in each partition, with partition 3 showing the highest stable state scEntropy, and partitions 7 and 0 showing the lowest (C). TICCI extrapolated HSMMs into a two-branched lineage model based on entropy, which contained two branches, 3→6→4→7 and 3→6→5→1→2→0, and the direction of the edge was determined by the stable state scEntropy of the two endpoints, from high to low. (D) HSMMs at single-cell resolution embedding. (E) Diffusion pseudotime results for HSMMs. (F) Multiple genetic marker expression patterns along the pseudotime pathway in 215 cells on the 3→6→5→1→2→0 branch mirror the dynamics in actual biological processes.

From Section 2, TICCI first identified four cell clusters from pretreated HSMMs and inferred an inter-cluster ligand–receptor communications network. Subsequently, CCI information was integrated into the cell neighborhood matrix using different weight parameters k. We applied 100 weighting parameters *k*, ranging from 0 to 10 in 0.1 increments. TICCI achieved optimal results for HSMM inference at *k* = 5.4. Here, “optimal” refers to the *k* value that maximizes the Pseudotime Accuracy Score (PAS), as defined in Section 3.3. TICCI generated an abstract map with eight cell partitions (Fig. 3A) and calculated stable state scEntropy for each partition to establish their sequential order in differentiation (Fig. 3B). Consistent with previous studies (Guo *et al.* 2017), TICCI was used to construct two-branched differentiation trajectories for HSMMs: 3→6→4→7 and 3→6→5→1→2→0 branches (Fig. 3C). Partition 3, consisting solely of proliferating cells, served as the starting point for differentiation. The 3→6→4→7 branch represented a process where proliferating cells were influenced by interstitial mesenchymal cells, while the 3→6→5→1→2→0 branch represented proliferating cells differentiating into myoblasts. Finally, TICCI mapped HSMMs at single-cell resolution (Fig. 3D) and estimated cell pseudotime using DPT (Fig. 3E).

To verify biological information in TICCI-inferred branches and trajectories, we examined key genetic marker expression patterns along the pseudotime pathway in HSMMs (Fig. 3F). Selected myogenic differentiation markers included: *CDK1*, *CCNA2*, *CCNB2*, *MYOG*, *MEF2C*, *CKM*, *ENO3*, *TNNT3*, and *MYH3*. *CCNA2*, *CCNB2*, and *CDK1* regulate the cell cycle. *MYOG* and *MEF2C* are required for skeletal muscle development and differentiation. Other molecules related to metabolism and exercise adaptation in muscle tissue, such as *CKM* and *ENO3*, also have significant roles. *CKM* transfers phosphate groups during muscle contraction, and *ENO3* catalyzes ATP production, a crucial step in glycolysis. *MYH3* encodes the primary structural protein in muscle fibers, which is essential for contraction. TNNT3 regulates calcium ions in muscle, crucial for contraction and relaxation. Thus, correct marker expression is vital for muscle health and growth (Watt and Driskell 2010). The inferred expression patterns of these key markers by TICCI were consistent with previous studies (Trapnell *et al.* 2014, Guo *et al.* 2017) showing that *CCNA2*, *CCNB2*, and *CDK1* levels decreased along the differentiation course, while *MYOG*, *MEF2C*, *CKM*, *ENO3*, *TNNT3*, and *MYH3* levels increased, thereby confirming trajectory veracity as deduced by TICCI.

### 3.2 TICCI infers AT2 trajectories

**Figure 4.**
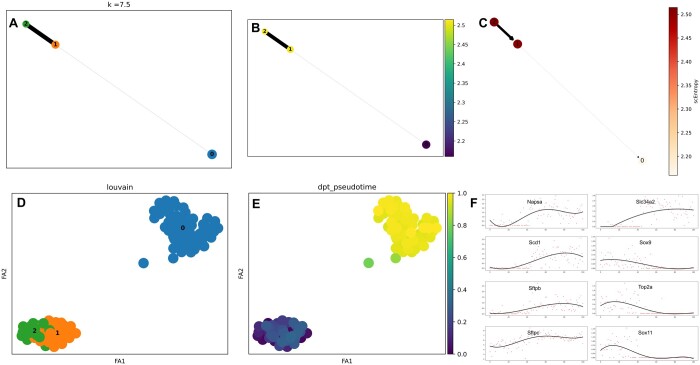
TICCI inferred trajectories in mouse alveolar type 2 (AT2) cells. (A) TICCI with an optimal CCI weight of 7.5 abstracted AT2 cells into three partitions, with thicker connecting lines between partitions indicating higher expression similarity. (B) To determine the differentiation path, TICCI calculated the steady state entropy of each partition; partition 2 had the highest steady state entropy and partition 0 had the lowest. (C) TICCI extrapolated AT2s into a single-branch lineage model based on entropy, which contained one branch, 2→1→0, and the direction of the edge was determined by the stable state scEntropy of the two endpoints, from high to low. (D) AT2 cells at single-cell resolution embedding. (E) Diffusion pseudotime results for AT2 cells. (F) Multiple genetic marker expression patterns along the pseudotime pathway in 101 cells at the 2→1→0 branch, consistent with biological developmental processes and previous findings.

Following Guo *et al.* (2017), we selected cells categorized as “AT2,” “Sftpc+,” and “Sftpc+Scgb3a2+” (*n* = 101) that showed gene expression in at least 30% of cells. Genes meeting these criteria were normalized and transformed using log2 transformation. Similar to the HSMM dataset, TICCI was used to cluster AT2 cells, infer intercellular ligand–receptor communication networks, and construct CCI information. This information was then multiplied by 100 different weighting parameters k (ranging from 0 to 10 in 0.1 increments) to integrate them into a cellular neighborhood matrix. TICCI achieved the optimal results for AT2 cell inference at a weighting parameter *k *=* *7.5. Here, “optimal” refers to the k value that maximizes the Pseudotime Accuracy Score (PAS), as defined in Section 3.3. TICCI also generated an abstract map with three cell partitions (Fig. 4A) and calculated stable state scEntropy for each partition to determine their sequential order during differentiation development (Fig. 4B). Aligning with previous studies (Treutlein *et al.* 2014, Guo *et al.* 2017), TICCI reconstructed a single-branch lineage model for AT2 cells: 2→1→0 (Fig. 4C), reflecting cell development from E14.5 to adult stages. TICCI further mapped AT2 cells at single-cell resolution embedding (Fig. 4D), and pseudotime was estimated using DPT (Fig. 4E).

To confirm biological information within branches and trajectories inferred by TICCI, we examined significant genetic marker expression patterns along the proposed chronological order in trajectory pathways constructed by TICCI for HSMMs (Fig. 4F). Several known myogenic differentiation marker genes were chosen for validation, including *CDK1*, *CCNA2*, *CCNB2*, *MYOG*, *MEF2C*, *CKM*, *ENO3*, *TNNT3*, and *MYH3*. Among these, CCNA2 and CCNB2 cytokines, along with the cell cycle protein-dependent kinase CDK1, are crucial during cell cycle regulation. The myogenesis factor MYOG and the myocyte enhancer factor MEF2C have vital roles in skeletal muscle development and differentiation. In addition, molecules related to cell metabolism and exercise adaptation, such as the muscle creatine kinase CKM and enolase ENO3, were identified. CKM is involved in transferring phosphate groups during muscle contraction, while ENO3 catalyzes ATP production from glycerol triphosphate to pyruvate, a key glycolysis step. Myosin heavy chain MYH3, essential for muscle contraction, encodes the main structural protein forming muscle fibers. Troponin TNNT3, which regulates calcium ions in muscle, is linked to muscle contraction and relaxation. Thus, correct protein expression is crucial for muscle health and growth (Watt and Driskell 2010).

These gene marker expression patterns along the trajectory inferred by TICCI were consistent with previous studies (Trapnell*et al.* 2014, Guo *et al.* 2017). *CCNA2*, *CCNB2*, and *CDK1* expression levels decreased as the proposed temporal order progressed, while *MYOG*, *MEF2C*, *CKM*, *ENO3*, *TNNT3*, and *MYH3* expression levels increased. This correlation suggested that trajectory branching inferred by TICCI reflected accurate biological developmental processes.

### 3.3 Methodological evaluation indicators

Dynverse (Saelens*et al.* 2019) provides a set of online guides for users to select the most suitable trajectory inference method for their dataset, which compares different trajectory inference methods. Following the dynverse concept, we proposed a Pseudotime Accuracy Score (PAS) metric to assess the accuracy of cell trajectory inference methods in real datasets. The main concept was to use external information such as cell type, cell sampling time, etc., which reflected real differentiation and developmental sequences in cells, to assess how well inferred pseudotemporal ordering matched external information. Notably, this external information was not used in trajectory inference processes, which was defined as follows:

$$\mathrm{PAS}=\sum_{i=1}^{N-1}\sum_{j>i}s(i,j)/M$$

 

si,j=0, T(i)=T(j)1/D, T(i)<T(j)-1/D, T(i)>T(j) 

where *N* denotes the number of all cells, s(i,j) denotes the accuracy score of pseudotime ordering of cell i with cell *j*, T(i) denotes the external information of cell *i*, *D* is the total number of two-by-two comparisons of all cells, and *M* is the theoretically maximal PAS based on external information and is used to normalize total scores. For each pair of cell *i* and cell *j* (*i* precedes *j* in the pseudotime order), the pseudotime order was considered consistent with external information if this information for cell *i* preceded that of cell *j*, i.e.$\;T\left(i\right)<T(j)$, then$\;s\left(i,j\right)=1$. If the external information of cell *i* was later than that of cell *j*, i.e.$\;T\left(i\right)>T(j)$, the pseudotime order was considered inconsistent with external information, then$\;s\left(i,j\right)=-1$. If external information of cell *i* and cell *j* was the same, then$\;s\left(i,j\right)=0\;$and PAS∈[-1,1]. When the PAS score was closer to 1, this indicated that pseudotime order in the cell trajectory better matched extrinsic information.

### 3.4 Parameter comparisons

**Figure 5.**
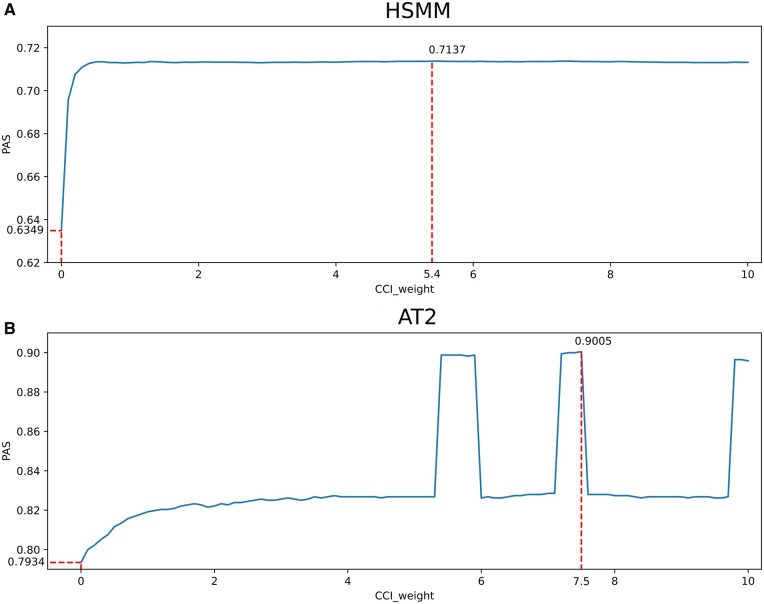
Comparing TICCI PAS values with different CCI weighting parameters in two real datasets. (A) For the HSMM dataset, and according to a previous study (Trapnell et al. 2014), as human skeletal muscle myoblast differentiation is completed from 0 to 48 h, the selection of cell collection time labels was no longer appropriate, and for this reason, the cell type label was used as a judgment criterion. Specifically, if the pseudotime of a proliferating cell was smaller than the pseudotime of an interstitial mesenchymal cell or a differentiating myoblast, the pseudotime was consistent with external information, while vice versa was wrong. The lowest TICCI PAS value in the HSMM dataset was 0.6349 when no CCI information was added, and the highest PAS value was 0.7137 when the CCI weight parameter was 5.4. (B) For the AT2 dataset, we used cell time labeling as a judgment criterion to reflect cell development stages, specifying that the correct pseudotime order should be consistent with E14.5, E16.5, E18.5, and adult stages. The results show that TICCI has the lowest PAS on the AT2 dataset at CCI weight parameter 0, which is only 0.7934, and the highest PAS at CCI weight parameter 7.5, which is 0.9005. These data showed the tangible enhancement effects of intercellular communication information on cell trajectory inference accuracy.

To determine the optimal CCI weight parameter k when joining the cell neighborhood matrix, we used 100 different weight parameters k covering the range from 0 to 10, taking values one by one in 0.1 increments, applied the TICCI algorithm to the HSMM and AT2 datasets, and compared time-accurate PAS values. Comparison results are shown (Fig. 5); When CCI information is unavailable, as shown in Fig. 5, TICCI operates with a cci_weight set to 0, effectively bypassing CCI data in trajectory reconstruction and pseudotime estimates. Under these conditions, the pseudotime accuracy score (PMS) is significantly lower when compared to scenarios where CCI data are used. This finding highlights a critical role for CCI information in enhancing the accuracy and robustness of TICCI’s trajectory inference.

As shown (Fig. 5), TICCI PAS values were evaluated using varying CCI weight parameters across HSMM and AT2 datasets. For the HSMM dataset, using time labels for cell collection was inappropriate due to completed myoblast differentiation within 48 h. Instead, cell-type labels were used for judgment. Specifically, a pseudotime score was considered correct if the proliferating cell's pseudotime was lower than that of an interstitial mesenchymal cell or differentiating myoblast, and otherwise incorrect. We observed that without CCI information, TICCI's PAS value was the lowest at 0.6349. The highest PAS value (0.7137) was achieved with a CCI weight parameter of 5.4, indicating that appropriate weighting significantly improved pseudotime accuracy.

For the AT2 dataset, cell label times, reflecting developmental stages (E14.5, E16.5, E18.5, and adult) were used as judgment criteria for pseudotime order accuracy. TICCI's lowest PAS value in this dataset was 0.7934 when the CCI weight parameter was 0. The highest PAS value (0.9005) was reached with a CCI weight parameter of 7.5. Thus, incorporating CCI information enhanced cell trajectory inference accuracy, as higher CCI weight parameters consistently improved PAS values.

These findings illustrated the importance of optimizing CCI weighting in pseudotime analysis. PAS improvements with increased CCI weight parameters across both datasets underscored the significant roles of intercellular communications in accurately determining cell trajectories. Thus, TICCI, with carefully adjusted CCI weights, is a robust method for cell trajectory inference, aligning pseudotime estimates more closely with actual biological processes.

### 3.5 Methodology comparisons

To further validate TICCI effectiveness in pseudotime analysis, we compared and evaluated TICCI with several other cellular trajectory inference algorithms: PAGA, SLICE, TSCAN, Monocle2, and Monocle3.

PAGA is a cell trajectory inference algorithm based on a partitioned graph abstraction method, which uses an approximate critical search method to represent cell relationships. The method uses a community discovery algorithm to construct an abstraction graph to determine lineage branching and uses DPT to calculate pseudotime values in cells (Wolf *et al.* 2019).

TSCAN (Ji and Ji 2019) is an minimum spanning tree (MST)-based cell trajectory inference algorithm, which uses PCA to reduce data dimensionality, then uses an EM (Expectation-Maximization, an iterative algorithm used for finding maximum likelihood estimates of parameters in probabilistic models, especially when the data is incomplete or has hidden variables) algorithm to cluster cells. The method then uses Bayesian Information Criterion to determine cluster numbers and constructs an MST with the cluster center as the node and infers the trajectory by determining the longest path of the tree. Finally, cells are projected onto the trajectory to generate the pseudotime order (Ji and Ji 2019).

Monocle2 is an improved version of original Monocle software; its most significant improvement is using DDRTree (an algorithm for simultaneously performing dimensionality reduction and learning tree structures from high-dimensional data) to iteratively find the location of cell projections in low-dimensional spaces in the cell lineage reconstruction process. Thus, Monocle2 more accurately determines cell position in low-dimensional spaces, and better characterizes relationships between cells and evolutionary trajectories. In low-dimensional spaces, Monocle2 uses the minimum spanning tree MST to represent lineage structures, which generates more accurate results (Qiu *et al.* 2017). Monocle3 is an improved version of Monocle2 and is mainly represented by the following aspects: (1) Support for dimensionality reduction using UMAP. When compared with the t-SNE algorithm (a technique for reducing dimensionality while preserving local data structure) used in Monocle2, under large-scale data, UMAP calculates cell positions in low-dimensional spaces faster, and better preserves local structures between cells; (2) A generated PAGA diagram shows relationships between cells, and identifies unconnected trajectories, which more accurately describe cell differentiation and evolutionary routes; (3) The user interaction interface is better optimized, with simpler and more intuitive operations, and more convenient parameter adjustments (Wolf *et al.* 2019).

**Figure 6.**
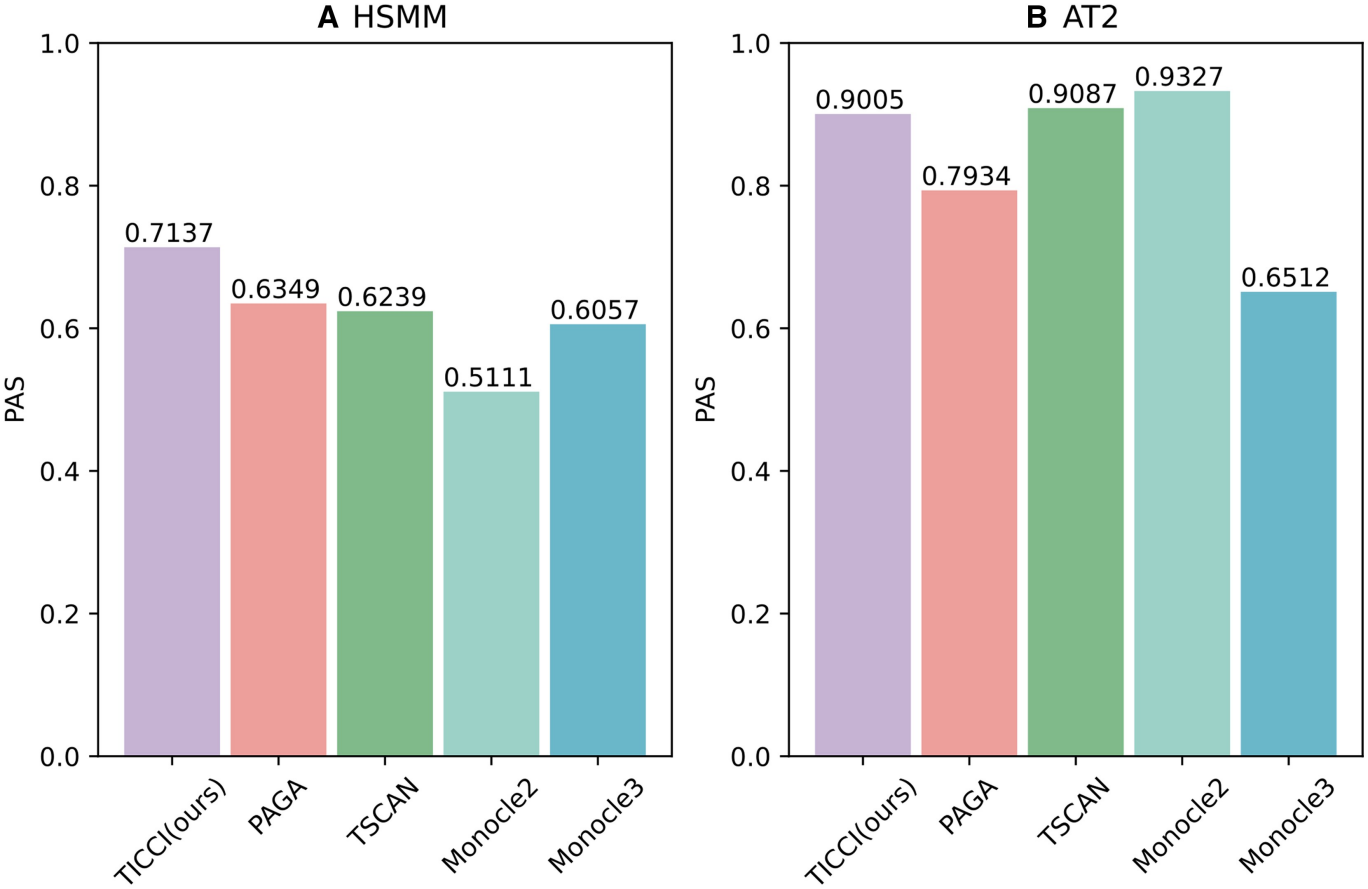
Comparing PAS values across different trajectory inference methods in two datasets. (A) For the HSMM dataset, TICCI achieved the best PAS. (B) For the AT2 dataset, the TICCI PAS was approximately equal to TSCAN, slightly inferior to Monocle2, but outperformed the other methods.

As shown (Fig. 6), trajectory inference methods were compared based on their PAS values in HSMM and AT2 datasets. A PAS score closer to 1 suggested that the proposed cellular time of the trajectory matched extrinsic information to a higher degree. For the HSMM dataset, TICCI achieved the highest PAS value, thus it was the most accurate in aligning pseudotimes with external biological signals. In contrast, for the AT2 dataset, TICCI's performance was approximately equivalent to TSCAN, slightly lower than Monocle2, but better than the other methods. These performance comparisons suggested that TICCI consistently provided reliable pseudotime estimates, although its relative performance varied depending on the dataset.

## 4 Discussion

Cell differentiation dynamics are fundamental to developmental biology. While bulk RNA-seq is a powerful high-throughput method that simultaneously profiles gene expression across hundreds to tens of thousands of cells or tissues, its reliance on large cell populations obscures intercellular heterogeneity. This heterogeneity, critical for understanding cell differentiation, developmental processes, and regulatory mechanisms, exists across tissue types and within subpopulations in the same tissue. In contrast, scRNA-seq enables researchers to resolve these differences at the single-cell level, providing unprecedented insights into cell-specific gene expression dynamics. ScRNA-seq has transformed molecular biology by elucidating intracellular activities, clarifying differentiation pathways, and identifying key gene functions during development. Inferring such cell differentiation trajectories based on scRNA-seq data is key to investigating changing cell patterns in different diseases. Several cell trajectory inference algorithms have been proposed which generally use similar gene expression levels between cells to construct cell differentiation trajectories and sort cells along differentiation trajectories to investigate cell differentiation and development processes. However, these algorithms face multiple challenges, such as high scRNA-seq data dimensionality, high noise, and a need for users to possess certain biological information about the datasets to effectively utilize the algorithms. In additional, intercellular communication information has important regulatory roles in cell differentiation and developmental processes, but current Trajectory Inference algorithms generally lack such information.

To address these issues, we proposed TICCI, a new method which reconstructs lineages on scRNA-seq data by combining intercellular communication information, which allows trajectory inference algorithms to generate results by considering more comprehensive biological information. TICCI uses a partitioned graph abstraction algorithm to identify trajectory topology and uses scEntropy to determine trajectory roots and directions without relying on user's knowledge of the dataset. TICCI also uses an improved DPT algorithm to estimate pseudotime in a cell.

Using TICCI, we successfully reconstructed cell trajectories in two real and two simulated scRNA-seq datasets. Lineage model and gene marker expression data along with cell trajectories were consistent with real cell differentiation and developmental processes, which reliably confirmed TICCI trajectories. We then verified that intercellular communication information exerted tangible enhancement effects on cell trajectory inference accuracy by using real cell differentiation stage labels as a benchmark. Finally, we compared TICCI with several popular trajectory inference methods on two real datasets.

For the HSMM dataset with a two-branch structure, TICCI achieved the best pseudotime-ordering accuracy. For the single-branch AT2 dataset, Monocle2 achieved the highest accuracy, while TICCI's score was slightly below the best result. We speculate that this was because AT2 was a small dataset with a simple single-branch structure. Methods such as TSCAN and Monocle, which use straightforward computational approaches, may be more effective for such datasets. In contrast, TICCI leverages random walk-based DPT and integrates multimodal biological information to infer trajectories, making it potentially better suited for datasets with more complex structures. In practical applications, users should try different trajectory inference methods to identify the one that best suits their specific needs. We also tested the proposed pseudotime estimates using only entropy or only CCI on two real datasets to demonstrate the superiority of the combined use. The experimental results are described in the Supplementary File.

The main TICCI parameters include: a threshold showing effective ligand–receptor gene expression; the dimension selected for PCA dimensionality reduction of high-dimensional gene expression data; the number of neighbors selected by proximity searches for constructing a class KNN graph, CCI weight parameters, and image abstraction resolution. In the log transformation of smoothing preprocessed data, the effective ligand–receptor gene expression threshold was set to 1. From scanpy default parameters, the dimensions selected for PCA was 50, and the neighbors selected for UMAP proximity searches was 10. The CCI weight parameter, which is the most important TICCI parameter, was compared between datasets in the range of 0 to 10, with an increment value of 0.1. We compared result scores from 100 CCI weight parameters between datasets, ranging from 0 to 10 in 0.1 incremental steps (see the Section 3.4). For cellular KNN maps with different weights added to CCI, we take different resolutions for map abstraction to obtain the cellular topology, the lower the resolution the smaller the number of partitions in the PAGA map, the less clear the embodied cellular topology, conversely too high a resolution may lead to some small partitions with too small a number of cells being too much affected by the extremes of the differentiation state when measuring the differentiation state according to the entropy. For the HSMM dataset, when using an optimal CCI weight parameter of 5.4, we selected a resolution of 1.4 to successfully construct a two-branched lineage structure. For the AT2 dataset, when using an optimal CCI weight of 7.5, we selected a resolution of 1.

Several cell communication analysis methods have been proposed: CellphoneDB (Efremova *et al.* 2020), Nichenet (Browaeys *et al.* 2020), and CellTalkDB (Shao *et al.* 2021). We used CellChat to compute communication patterns between cell subpopulations; however, researchers can select different methods or use them in combination to better fit their datasets. In addition, several methods similar to scEntropy also use the entropy concept to measure differentiation status in cells, such as signal entropy (Banerji *et al.* 2013) and StemID (Grün *et al.* 2016). We selected scEntropy as it successfully used expression patterns from a larger portion of the transcriptome for entropy calculations, thus reducing information loss during calculations. However, these methods are complementary, and for individual studies, researchers should select the most appropriate methods to apply on their research.

Our study has some limitations that warrant further exploration. Specifically, the Chu–Liu algorithm used in our method to reconstruct trajectory branches does not recognize cyclic trajectory structures. In addition, our implementation scope for TICCI is currently restricted to human and mouse cells due to its reliance on ligand–receptor interaction datasets provided by CellChat.

Looking ahead, a promising direction for future research is to leverage spatial transcriptomic data to distinguish between short- and long-range intercellular communication relationships. By integrating multimodal biological information in trajectory inference, we can reduce biological noise caused by sampling with a single technology, thus enhancing the accuracy and comprehensiveness of our results. In addition, another potential benchmark for evaluating trajectory inference methods like TICCI is to use genealogical information, such as somatic mutations, to determine the exact topology of cell lineage trees. This approach could complement current pseudotime validation strategies by providing direct lineage insights. Incorporating this approach in future exploration studies could provide additional validation of inferred trajectories, providing deeper insights into lineage relationships and enhancing the robustness of trajectory inference methods.

In conclusion, we propose TICCI, a heuristic experimental approach for reconstructing cell trajectories from scRNA-seq data. TICCI outperforms existing trajectory inference methods and further demonstrates its advantages on real datasets. TICCI incorporates intercellular communication information to generate more accurate results that consider more comprehensive biological information and allow for the determination of trajectory roots and paths without requiring users to have extensive biological knowledge of the datasets. In future research, we will improve TICCI and apply it to other biological processes.

## Supplementary Material

btaf027_Supplementary_Data

## Data Availability

Source code and binaries freely available for download at https://github.com/mine41/TICCI.
