# Supplementary material for: Trajectory Inference with Cell–Cell Interactions (TICCI): intercellular communication improves the accuracy of trajectory inference methods
Source: Bioinformatics. 2025 Feb 3;41(2):btaf027. doi: 10.1093/bioinformatics/btaf027 (PMC11829803; doi:10.1093/bioinformatics/btaf027)
Supplement: btaf027_Supplementary_Data [file btaf027_supplementary_data.zip › 40fc4_Supplementary file.docx]

**Supplementary experiment 1**

We apply TICCI to two simulated datasets generated by scMultiSim. The first dataset consists of 100 cells organized into a phylogenetic tree structure with 3 tips and 2 internal nodes (Figure S1 A), hereafter referred to as Phyla3. The second dataset consists of 300 cells organized into a phylogenetic tree structure with 5 tips and 4 internal nodes(Figure S2 A). Both datasets use other parameters: GRN = GRN_params_100, num.cif = 20, discrete.cif = F, cif.sigma = 0.1, Parameter usage are described in scMultiSim's Parameter Guide. In order to simulate the intercellular communication carried out by similar cells, we selected the genes with the highest expression in the top 1% of neighboring cells as the communication ligand and receptor to construct the database, and the communication was constructed randomly using the parameter cell.type.interaction = “random”.

As with the approach in the main text, TICCI generated an abstract map on Phyla3 with five cell partitions (Fig. S1 C). Since the genes of the simulated data do not correspond to real cellular functions, it was not possible to calculate the scEntropy of the simulated cells to identify the root cell as well as the branching direction, so we manually selected the cell with the smallest depth as the root cell. Finally, TICCI mapped Phyla3 at single-cell resolution ( Figure S1 D) and estimated cell pseudotime using DPT (Figure S1 E).


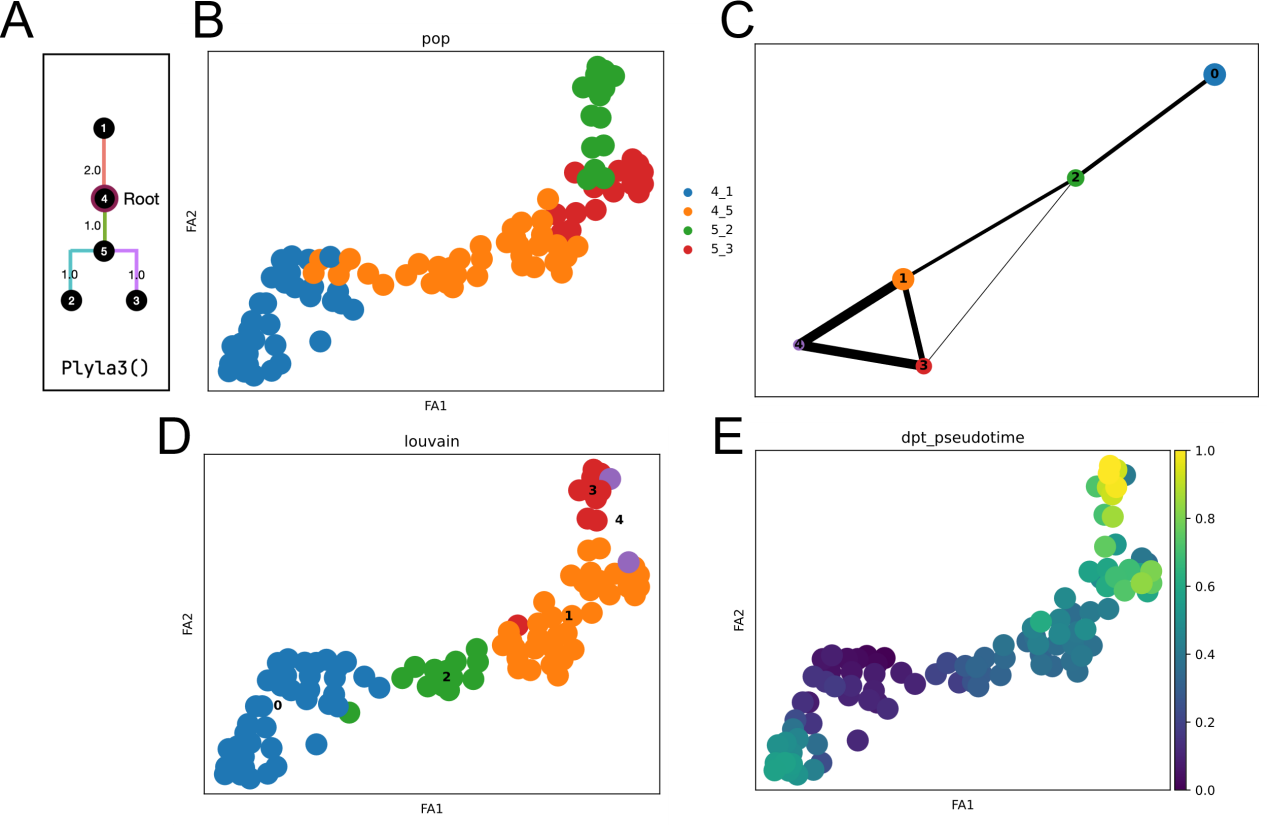


**Figure S1. TICCI-Inferred Trajectories in the Simulated Phyla3 Dataset.**

(A) Schematic representation of the structure of the Phyla3 dataset.

(B) ForceAtlas2 visualization of the Phyla3 dataset.

(C) TICCI's abstraction of Phyla3 into five partitions, where connecting line thickness indicates the degree of expression similarity between partitions.

(D) Single-cell resolution embedding of the Phyla3 dataset.

(E) Diffusion pseudotime results for the Phyla3 dataset.

TICCI generated an abstract map on Phyla5 with 13 cell partitions (Fig. S2 C). Since the genes of the simulated data do not correspond to real cellular functions, it was not possible to calculate the scEntropy of the simulated cells to identify the root cell as well as the branching direction, so we manually selected the cell with the smallest depth as the root cell. Finally, TICCI mapped Phyla5 at single-cell resolution ( Figure S2 D) and estimated cell pseudotime using DPT (Figure S2 E).


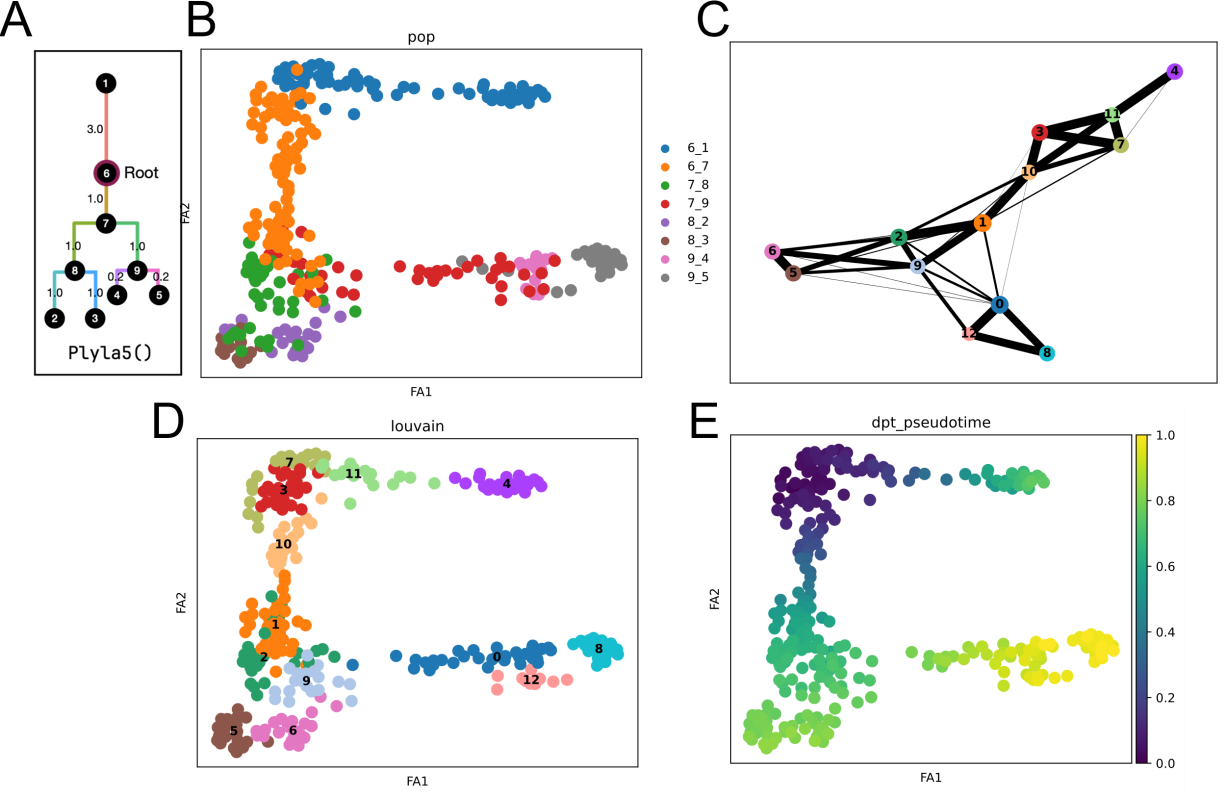


**Figure S2. TICCI-Inferred Trajectories in the Simulated Phyla5 Dataset.**

(A) Schematic representation of the structure of the Phyla5 dataset.

(B) ForceAtlas2 visualization of the Phyla5 dataset.

(C) TICCI's abstraction of Phyla5 into 13 partitions, where connecting line thickness indicates the degree of expression similarity between partitions.

(D) Single-cell resolution embedding of the Phyla5 dataset.

(E) Diffusion pseudotime results for the Phyla5 dataset.

To determine the optimal CCI weight parameter k when joining the cell neighborhood matrix, we used 100 different weight parameters k covering the range from 0 to 10, taking values one by one in 0.1 increments, applied the TICCI algorithm to two simulated datasets, and compared time-accurate PAS values. Comparison results are shown (Figure S3); Unlike the real datasets where real labels were used to obtain the PAS, each cell in the simulated dataset has a depth value that measures its distance from the root cell. We used the same degree of intercell pseudotime value and depth as PAS. PAS values obtained by adding CCI information to cell trajectory inferences with multiple parameters were all larger than those without CCI information (weighted at 0). This showed the tangible enhancement effects of intercellular communication information with respect to cell trajectory inference accuracy.


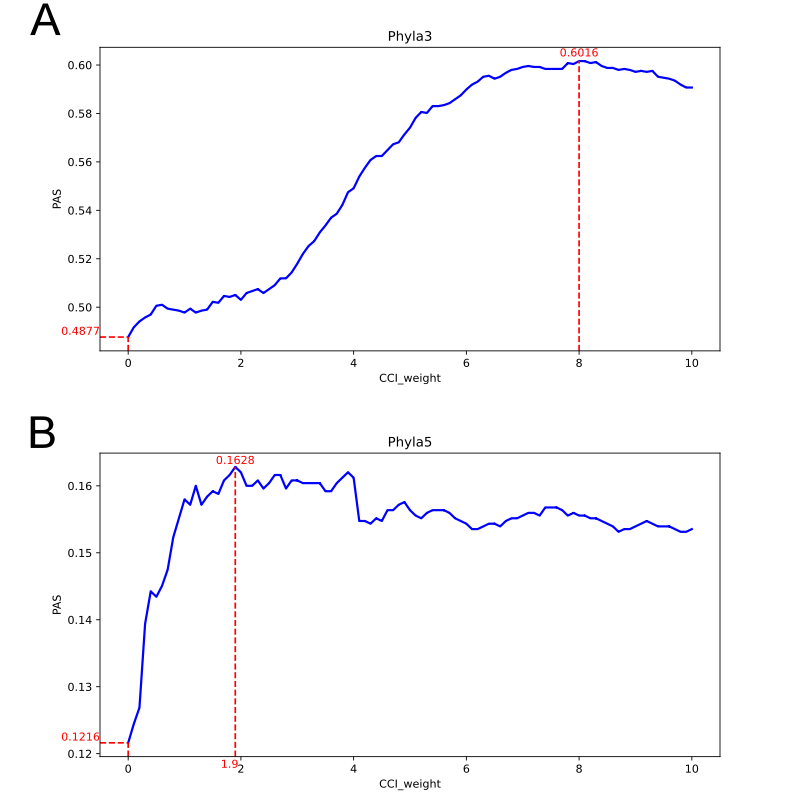


**Figure S3. Comparison of TICCI's Partition Assignment Score (PAS) with Different Cell Cycle Information (CCI) Weighting Parameters in Simulated Datasets.**

(A) For the Phyla3 dataset, the lowest PAS value of 0.4877 was obtained when the CCI weight parameter was 0, while the highest PAS value of 0.6016 was reached with a CCI weight parameter of 8.0.

(B) For the Phyla5 dataset, the lowest PAS value of 0.1216 was obtained when the CCI weight parameter was 0, while the highest PAS value of 0.1628 was reached with a CCI weight parameter of 1.9.

**Supplementary experiment 2**

To determine the effect of CCI or scEntropy alone, we performed ablation experiments on two real datasets. The approach was as follows: (1) Instead of using the cell similarity obtained by UMAP nearest neighbor search, only CCI was used to construct the cell similarity matrix. The pseudotime value was then estimated, hereafter referred to as “TICCI (only CCI)”. (2) The scEntropy of each cell was used as the pseudotime value to calculate the PAS, hereafter referred to as “only scEntropy”. The experimental results are shown in Figure S4, demonstrating the superiority of the combination.

It is worth discussing that “only scEntropy” scores significantly lower on HSMM, which we believe demonstrates the limitations of using only entropy. scEntropy’s measurement is derived from cellular functional activation levels, as reflected by gene expression in individual cells, which indicates each cell’s differentiation potential. Some Cells in the transitional phases have insufficient functional activation, this makes entropy insufficient for accurately reflecting their pseudotime ordering. Furthermore, cells in different differentiation branches activate distinct cellular functions, making it difficult to use entropy alone to describe their relative distance from the root cells.

**
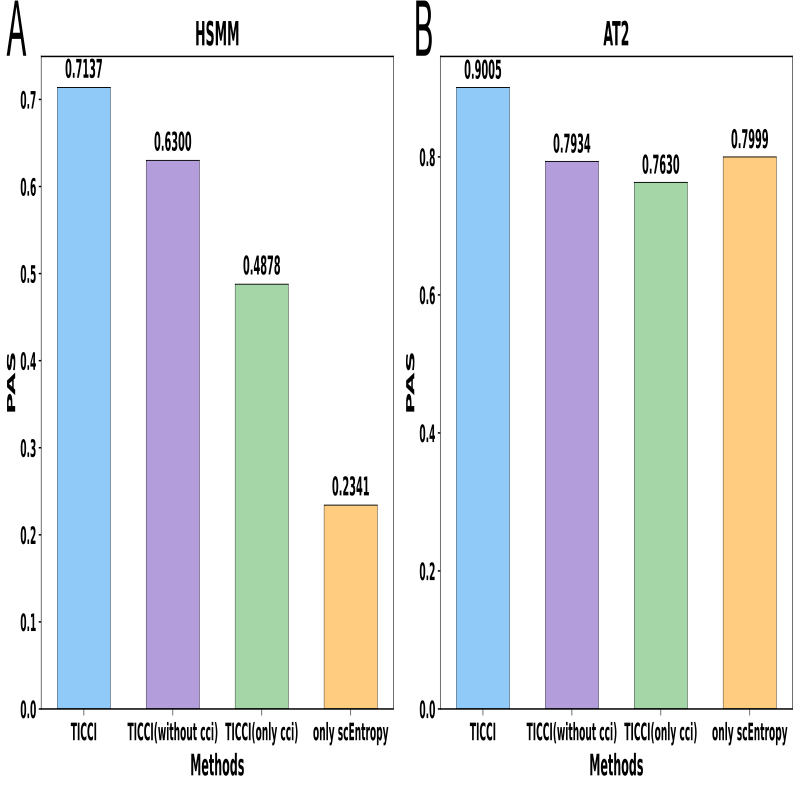
**

**Figure S4. Comparison of PAS Values Obtained by TICCI on Two Real Datasets Using Different Data Sources.** (A) For the HSMM dataset, the combination of all information sources results in the highest PAS value. In contrast, the PAS value is significantly lower when using only scEntropy (single-cell entropy) data. We hypothesize that this is because the HSMM trajectory has a two-branch structure. Cells in different differentiation branches activate distinct cellular functions, so entropy obtained from cellular functional activation levels may not accurately measure cell pseudotime values across diverging branches. (B) For the AT2 dataset, which has a single-branch trajectory, the combination of all data sources again achieves the highest PAS value. However, in this case, the approach utilizing only scEntropy also performs well, likely because the single-branch structure allows the entropy-based pseudotime estimation to effectively capture progression along the trajectory.
