# Supplementary figures and images for: Trajectory Inference with Cell–Cell Interactions (TICCI): intercellular communication improves the accuracy of trajectory inference methods
Source: Bioinformatics. 2025 Feb 3;41(2):btaf027. doi: 10.1093/bioinformatics/btaf027 (PMC11829803; doi:10.1093/bioinformatics/btaf027)

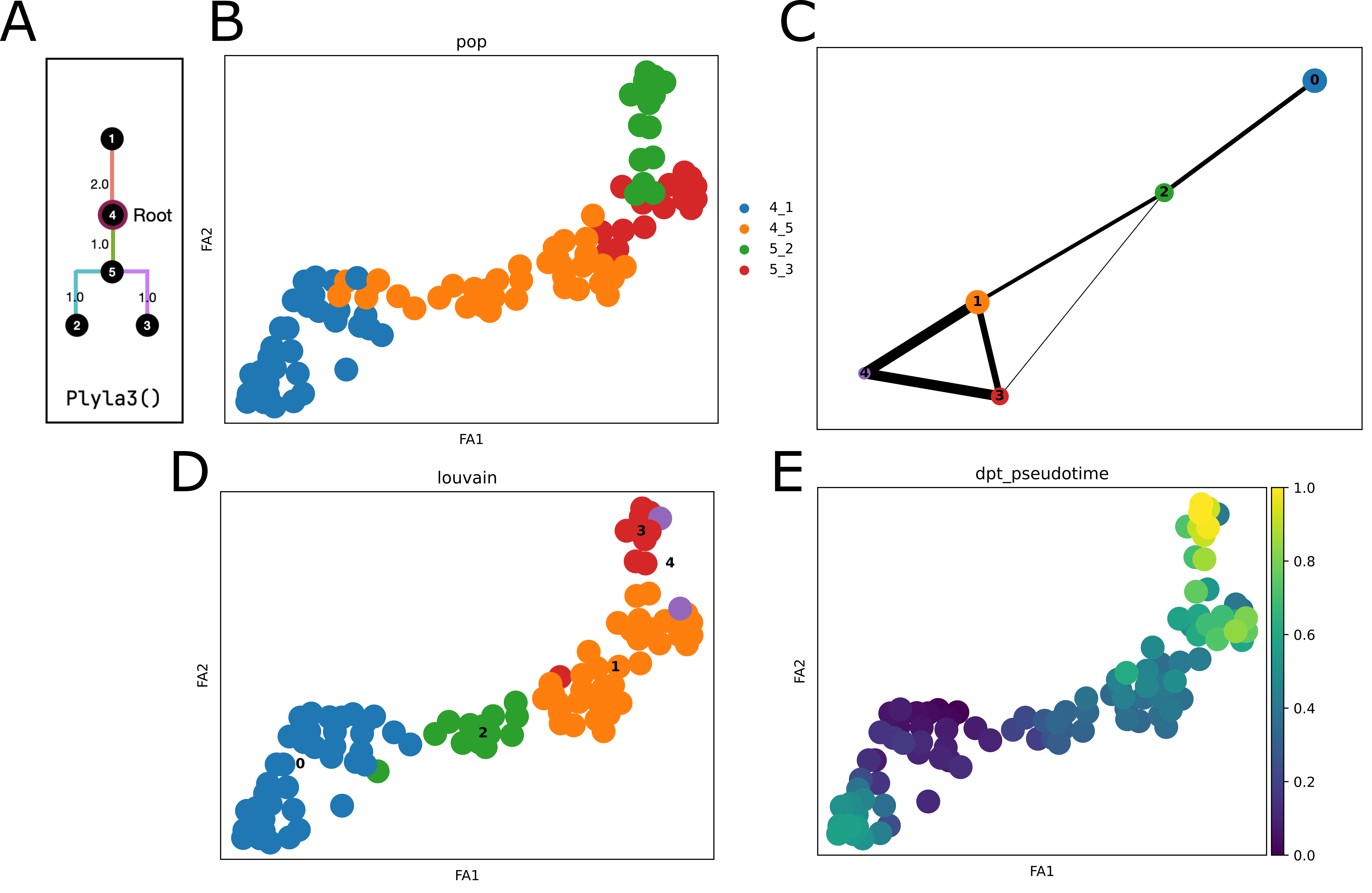

Supplement: btaf027_Supplementary_Data [file btaf027_supplementary_data.zip › 4b4a8_FigureS1.png]

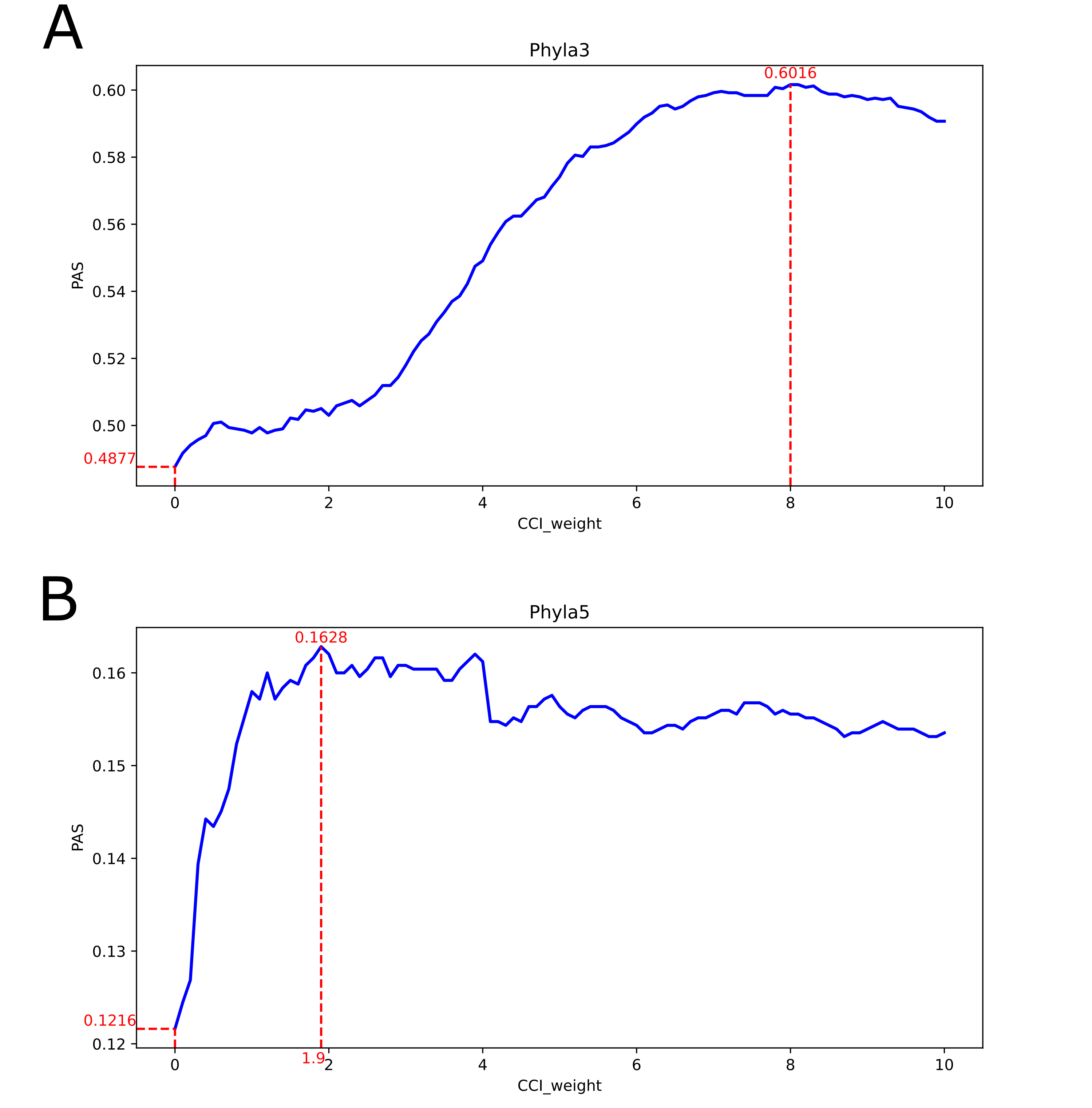

Supplement: btaf027_Supplementary_Data [file btaf027_supplementary_data.zip › 7b0e3_FigureS3.png]

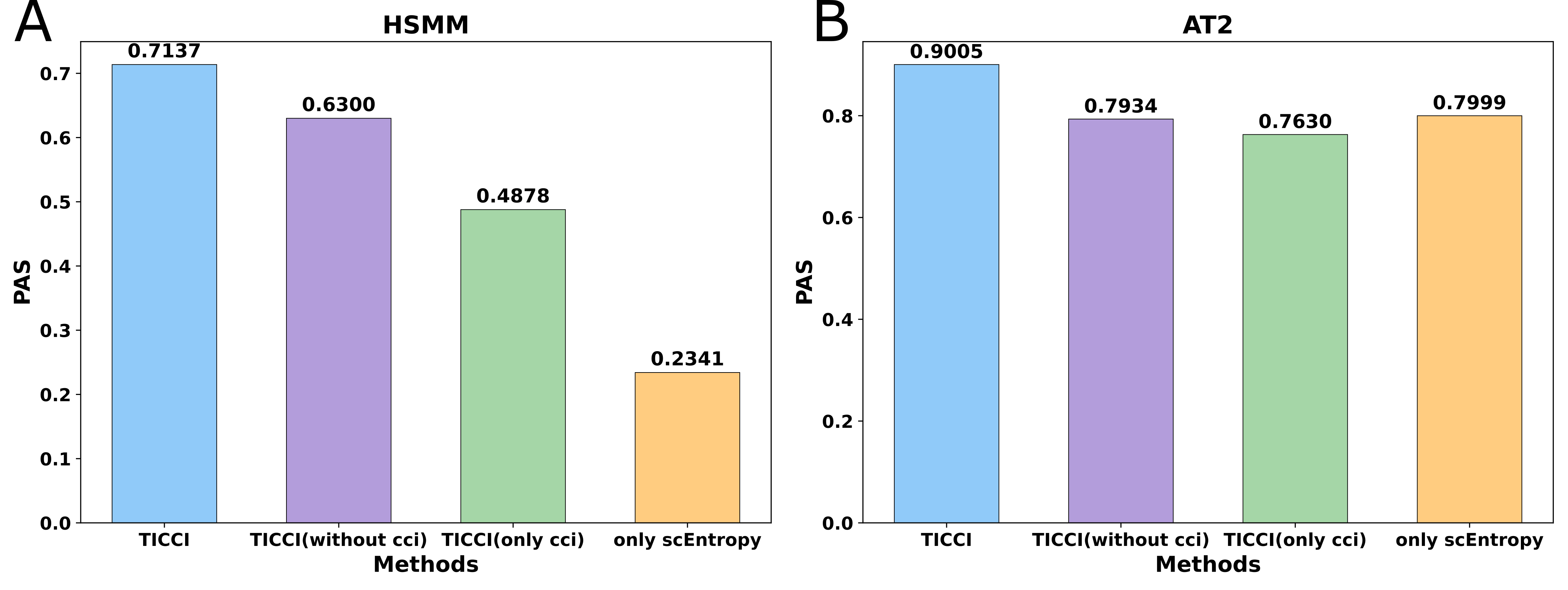

Supplement: btaf027_Supplementary_Data [file btaf027_supplementary_data.zip › e6d07_FigureS4.png]

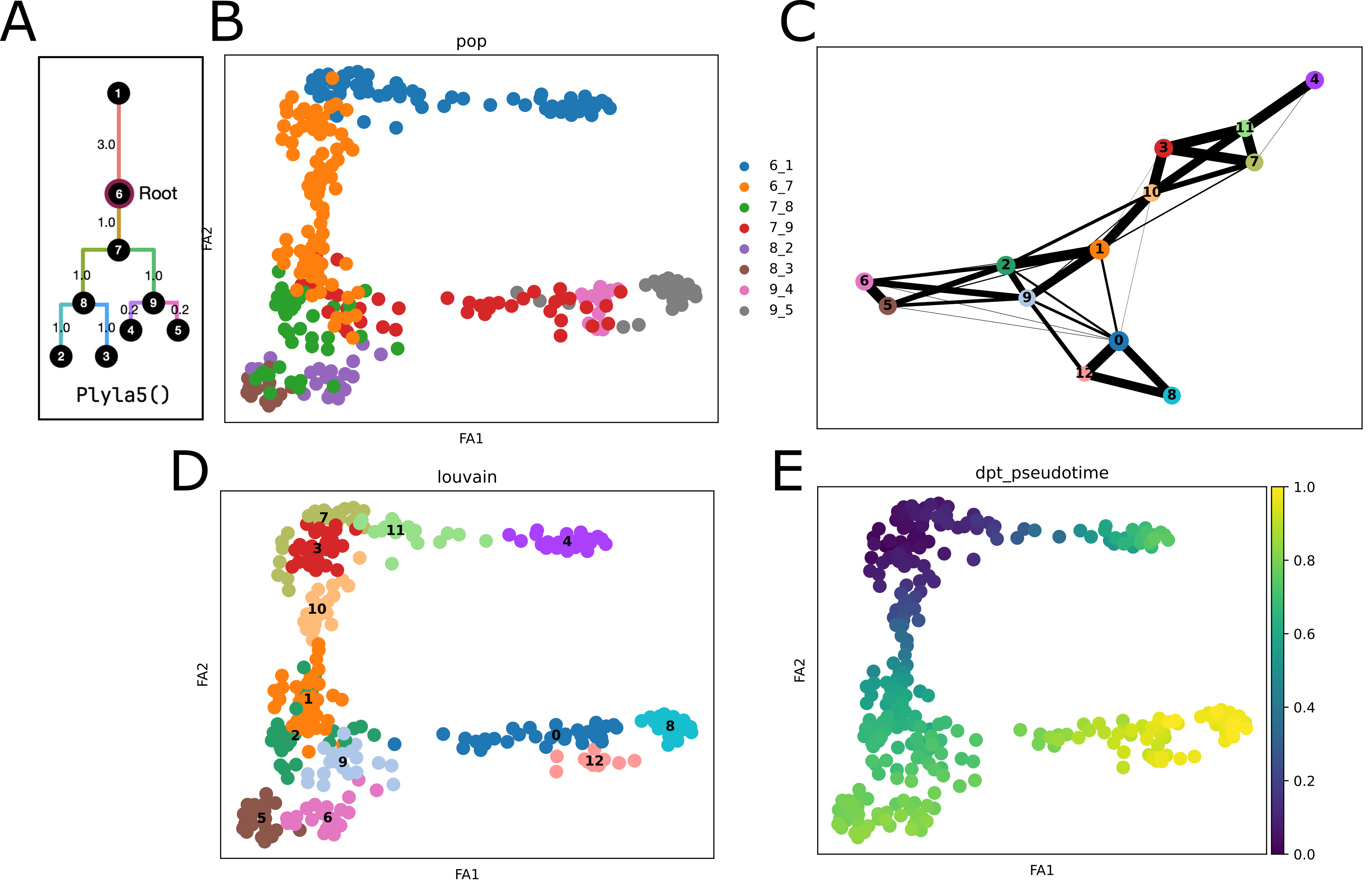

Supplement: btaf027_Supplementary_Data [file btaf027_supplementary_data.zip › 82983_FigureS2.png]
